# Beyond the Thrombophilia Panel: Real-World Overuse, Limited Interpretability, and Poor Guideline Concordance in a Hematology Referral Cohort

**DOI:** 10.3390/diagnostics16162643

**Published:** 2026-08-19

**Authors:** Aysenur Ozturk Ari, Ibrahim Ethem Pinar, Vildan Ozkocaman, Fahir Ozkalemkas

**Affiliations:** 1 Department of Internal Medicine, Faculty of Medicine, Bursa Uludag University, Bursa 16059, Türkiye; dr.aysenurozturk@gmail.com; 2 Department of Hematology, Faculty of Medicine, Bursa Uludag University, Bursa 16059, Türkiye

**Keywords:** hereditary thrombophilia, thrombophilia testing, factor V Leiden, lupus anticoagulant, guideline concordance, laboratory stewardship

## Abstract

**Background:** Hereditary thrombophilia testing is frequently performed outside guideline-supported indications, generating low-value results and diagnostic uncertainty. We evaluated the spectrum, laboratory validity, and appropriateness of thrombophilia testing in patients referred to hematology. **Methods:** This single-center retrospective cohort included 131 adults referred between September 2021 and November 2022 with a completed six-variant hereditary thrombophilia panel. Demographic, clinical, thrombotic, and laboratory data were reviewed, and testing appropriateness was assessed against the 2011 Turkish Society of Hematology guideline. **Results:** Ischemic stroke (25.2%), pulmonary embolism (21.4%), and recurrent pregnancy loss (16.0%) were the leading indications. Of 120 evaluable requests, only seven (5.8%) met guideline-supported criteria, and 42 (32.0%) were ordered during acute thrombosis. Factor V Leiden was associated with pulmonary embolism (48.6% vs. 22.1%, *p* = 0.003) and deep vein thrombosis (28.6% vs. 9.5%, *p* = 0.003), whereas ischemic stroke was less frequent among carriers (11.4% vs. 33.7%, *p* = 0.017). PAI-1, MTHFR c.1298A>C, and prothrombin G20210A showed no consistent associations. Of six low protein C results, only two were confirmed; none of eight low protein S results remained valid after timing and confirmation criteria were applied. Lupus anticoagulant was positive in 18 patients, yet only six underwent appropriately timed repeat testing, establishing four new antiphospholipid syndrome diagnoses. **Conclusions:** Most thrombophilia panels were ordered inappropriately, and test timing frequently compromised interpretability. Clinically meaningful information arose mainly from factor V Leiden and properly confirmed acquired thrombophilia testing. Targeted, indication-driven testing with strict attention to timing and confirmation should replace indiscriminate panel use.

## 1. Introduction

Thrombophilia denotes an increased predisposition to thrombosis arising from inherited or acquired abnormalities of hemostasis. Established inherited thrombophilias include factor V Leiden (FVL), the prothrombin G20210A variant, and deficiencies of antithrombin, protein C, and protein S. Although these defects are linked primarily to venous thromboembolism (VTE), their detection rarely establishes the cause of an individual event or mandates a change in treatment. For most patients, the duration of anticoagulation is determined more by whether the event was provoked or unprovoked, the balance of recurrence and bleeding risk, and patient preference than by the presence of a common inherited thrombophilia [1,2,3].

Contemporary guidelines have therefore shifted away from unrestricted screening toward selective testing reserved for situations in which the result is expected to alter management. The 2023 American Society of Hematology guideline discourages routine testing in many common VTE scenarios, particularly after surgery-provoked events or when anticoagulation would continue regardless of the result. Testing may be considered in selected patients with VTE provoked by hormonal or nonsurgical transient factors, in cerebral or splanchnic vein thrombosis when stopping treatment is otherwise planned, or when a strong family history of high-risk thrombophilia could influence prophylaxis, pregnancy, or estrogen-related decisions [2]. Testing during acute thrombosis or early anticoagulation further complicates interpretation of protein C, protein S, antithrombin, and lupus anticoagulant assays, yielding transient or falsely abnormal results [3].

Despite these recommendations, thrombophilia tests remain heavily ordered. Real-world studies consistently document high rates of inappropriate or low-value testing, with proportions varying by population, setting, and criteria [4,5,6]. Recurrent patterns include testing during acute thrombosis or anticoagulation, testing after clearly provoked VTE, and indiscriminate panel use in patients with arterial thrombosis, stroke, adverse obstetric outcomes, or no confirmed thrombotic event. Such practices can generate misleading diagnoses, unnecessary repeat testing, prolonged anticoagulation, anxiety, and avoidable cost without improving outcomes [1,4,5,6].

The value of inherited thrombophilia testing is especially uncertain outside venous presentations. In young adults with ischemic stroke, associations with inherited thrombophilias are inconsistent and abnormal results rarely change secondary prevention, so targeted evaluation for antiphospholipid syndrome is preferred [7]. Routine screening is likewise not recommended in recurrent pregnancy loss: the ALIFE2 randomized trial showed that low-molecular-weight heparin did not improve live-birth rates in women with recurrent miscarriage and confirmed inherited thrombophilia [8]. Panel composition is a further concern, since many panels add common polymorphisms—MTHFR c.677C>T and c.1298A>C, plasminogen activator inhibitor-1 (PAI-1) 4G/5G, and factor XIII—that lack a validated role in routine assessment [9,10,11,12].

Against this background, we aimed to characterize the clinical and thrombotic phenotypes of adults referred to hematology with completed thrombophilia panels, examine the distribution of tested variants and their associations with thrombotic manifestations, assess the timing and interpretability of additional assays, and determine the appropriateness of panel requests against the national guideline in force during the study period.

## 2. Materials and Methods

### 2.1. Study Design and Setting

This descriptive, single-center, non-interventional, retrospective cohort study was conducted in the Department of Hematology of Bursa Uludag University Faculty of Medicine. It had two objectives: to characterize the clinical spectrum of thrombotic disease among patients referred with a hereditary thrombophilia panel result, and to evaluate whether these requests conformed to accepted testing indications. During the study period, thrombophilia-panel testing was not restricted to the Department of Hematology and could be requested electronically by physicians from multiple specialties, without mandatory hematology preauthorization, indication-based restriction, or computerized decision support. For patients covered by the national health-insurance scheme, the panel was reimbursed by the Social Security Institution (SGK) under the applicable Health Implementation Communiqué (SUT) tariff and was not billed directly to the patient; the tariff was approximately TRY 286–314 per panel (time-weighted average TRY 296.49, ≈US$20.77 per request).

### 2.2. Participants

Consecutive adults referred between September 2021 and November 2022 with a completed panel result were eligible. Our hereditary thrombophilia panel comprises six molecular tests: FVL, the prothrombin G20210A variant (PGM), the MTHFR c.677C>T and c.1298A>C polymorphisms, PAI-1, and factor XIII. Patients aged at least 18 years with all six tests performed were included; those younger than 18 years or with unretrievable results were excluded. In total, 131 patients met these criteria. During the same period, a hereditary thrombophilia panel was performed in 554 adults across the institution; the 131 patients subsequently referred to hematology with a complete panel therefore represented 23.6% of all tested adults and constituted the study cohort.

### 2.3. Data Collection and Definitions

Demographic data and comorbidities (hypertension, diabetes mellitus, hyperlipidemia, malignancy, and rheumatologic disease) were abstracted from the hospital information system, together with acquired prothrombotic factors including immobilization, pregnancy, the postpartum period, smoking, myeloproliferative disease, antiphospholipid syndrome, nephrotic syndrome, oral contraceptive or hormone use, major surgery, indwelling catheters, cirrhosis, family history, and obesity. A positive family history was defined a priori as VTE or sudden death before 50 years in a first-degree relative, and obesity as a body mass index of at least 30 kg/m^2^. Age at first thrombosis, recurrence, anticoagulant use at sampling, and acute-versus-chronic phase were recorded; the acute phase was an arterial or venous event within the preceding 30 days. Radiologically confirmed events were classified by site, and patients were assigned to one of four phenotypes: arterial, venous, combined arterial and venous, or recurrent pregnancy loss (RPL). Recurrent pregnancy loss was defined, in accordance with European Society of Human Reproduction and Embryology guidance [13], as the loss of two or more pregnancies. Pregnancy losses following spontaneous conception or assisted reproductive technology were included, whereas ectopic and molar pregnancies and implantation failure were excluded. This clinical definition is broader than, and was applied separately from, the antiphospholipid-syndrome obstetric classification criterion.

### 2.4. Thrombophilia Testing and Laboratory Evaluation

Genetic testing was performed using the Thrombophilia Multiplex Real-Time PCR Kit A (Cat. No. 10R-20-08A; SNP Biotechnology R&D Ltd., Hacettepe Technopolis, Ankara, Türkiye). Gene tests were reported qualitatively (homozygous, heterozygous, or no mutation), and PAI-1 as 4G/4G, 4G/5G, or 5G/5G. The factor XIII variant genotyped was F13A1 NM_000129.4:c.103G>T (rs5985; NP_000120.2:p.Val35Leu), conventionally designated as factor XIII Val34Leu (V34L). Where available, protein C, protein S, and antithrombin activity; homocysteine and factor VIII; a paroxysmal nocturnal hemoglobinuria panel; and HLA-B51 status were collected. Antiphospholipid antibodies, homocysteine, and factor VIII obtained at diagnosis were interpreted according to the institutional reference ranges presented in Table 1. Lupus anticoagulant testing was performed using a dilute Russell viper venom time (dRVVT)-based assay on platelet-poor citrated plasma, comprising a screening step and a phospholipid-rich confirmatory step; a normalized screen-to-confirm ratio greater than 1.20, according to the institutional laboratory cutoff, was considered positive, and a separate lupus anticoagulant-sensitive aPTT-based assay was not routinely performed. A positive non-genetic result required confirmation at least 6 weeks later; for study classification, antiphospholipid syndrome required at least one qualifying clinical criterion together with persistent positivity for at least one criterion antiphospholipid antibody on at least two occasions separated by at least 12 weeks, in accordance with the revised Sydney classification criteria [14].

### 2.5. Assessment of Appropriateness and Statistical Analysis

Each request was assessed against the 2011 Turkish Society of Hematology guideline [15], the national standard throughout the study period (Table 2). Requests with a single documented indication were classified as appropriate or inappropriate; combined-RPL, undocumented, and family-screening requests were excluded from the denominator. The study was approved by the institutional Clinical Research Ethics Committee (6 December 2022; 2022-19/16) and followed the Declaration of Helsinki. Non-normally distributed continuous variables are summarized as median (range) and age additionally as mean ± SD; categorical variables as counts and percentages. Comparisons used the Mann–Whitney U, chi-square, or Fisher–Freeman–Halton test in IBM SPSS Statistics v23.0 (IBM Corp., Armonk, NY, USA), with two-sided *p* < 0.05 considered significant. Given the modest size of several subgroups, formal between-group comparisons in the main text were limited to the adequately sized factor V Leiden subgroup; the remaining phenotype and variant comparisons are reported descriptively as counts and percentages, and the full exploratory variant–phenotype analysis is provided in the Supplementary Materials.

## 3. Results

### 3.1. Patient Characteristics

Between September 2021 and November 2022, 131 patients were included. Women predominated (84, 64.1%). The mean age at first thrombotic event was 39.7 ± 11.6 years (range 18–70), and 72 patients (55.0%) sustained their first event by age 40. The most frequent comorbidities were hyperlipidemia (22.9%), obesity (21.4%), hypertension (20.6%), and diabetes mellitus (13.7%); malignancy (5.3%) and rheumatologic disease (3.8%) were uncommon (Table 3).

### 3.2. Indications and Ordering Specialties

**Figure 1:**
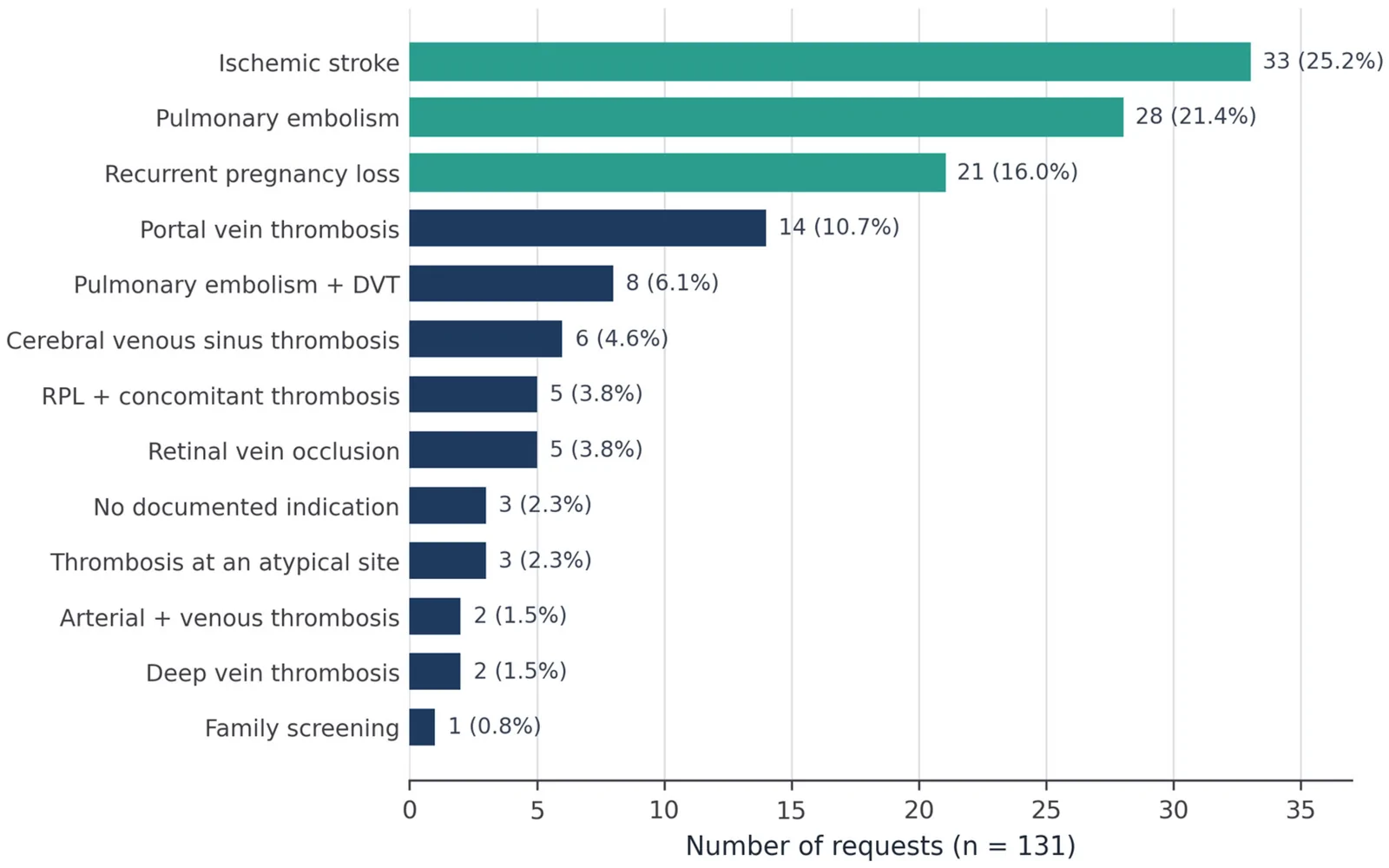
Indications for hereditary thrombophilia panel testing (*n* = 131). The three most frequent indications—ischemic stroke, pulmonary embolism, and recurrent pregnancy loss (RPL)—together accounted for 62.6% of all requests. Combined RPL categories are pooled. DVT, deep vein thrombosis.

Panels were requested by 15 specialties. Ischemic stroke was the single most common indication (25.2%, mostly from neurology), followed by pulmonary embolism (PE; 21.4%, mostly from chest diseases) and RPL (16.0%). Portal vein thrombosis (10.7%) and concurrent PE with deep vein thrombosis (DVT; 6.1%) followed, whereas cerebral venous sinus thrombosis (CVST; 4.6%), retinal vein occlusion (3.8%), and atypical-site thrombosis (2.3%) were less common. No indication was documented for 2.3% of requests, and 0.8% were ordered for asymptomatic family screening (Figure 1).

### 3.3. Thrombotic Phenotypes

Patients were grouped as arterial (*n* = 39), venous (*n* = 62), combined arterial and venous (*n* = 7), and recurrent pregnancy loss (*n* = 21). Because several of these groups were small, the phenotype comparisons are presented descriptively, without formal statistical testing. The distribution of sex and of age at first event was broadly similar across the arterial, venous, and combined groups. Hyperlipidemia was numerically more frequent in the combined group than in the recurrent pregnancy loss group (57.1% vs. 9.5%), and malignancy was recorded only in the venous (8.1%) and combined (28.6%) groups, being absent in the arterial and recurrent pregnancy loss groups (Table 4). Smoking status was available for 127 patients and was documented in 50 (39.4%); its distribution across the clinical phenotypes is presented descriptively in Table 5. The remaining acquired risk factors were similarly distributed across phenotypes (Table 5).

### 3.4. Distribution of Hereditary Thrombophilia Variants

The most frequent finding was the PAI-1 4G/5G genotype (48.9%), followed by heterozygous MTHFR c.677C>T (45.8%) and c.1298A>C (38.9%). Heterozygous FVL and PGM were detected in 26.9% and 13.7% of patients, respectively; homozygous FVL and PGM were each found in one patient (Table 6).

### 3.5. Association Between Variant Type and Thrombotic Manifestation

Among the panel variants, heterozygous factor V Leiden—an adequately sized subgroup (*n* = 35)—showed the clearest and biologically most coherent association: pulmonary embolism was more frequent among carriers than non-carriers (48.6% vs. 22.1%, *p* = 0.003), as was deep vein thrombosis (28.6% vs. 9.5%, *p* = 0.003), whereas ischemic stroke was less frequent among carriers (11.4% vs. 33.7%, *p* = 0.017). This prespecified comparison is retained because its subgroup is not subject to the small-sample concern; all other genotype subgroups were small, and their comparisons are therefore reported descriptively and regarded as exploratory. The remaining variants showed no consistent relationship with any manifestation. The rare homozygous findings are described as individual observations rather than statistical results: the single patient with homozygous factor V Leiden had both pulmonary embolism and deep vein thrombosis; the single patient with homozygous prothrombin G20210A presented with recurrent pregnancy loss; and cerebral venous sinus thrombosis occurred in two of the five patients homozygous for F13A1 Val34Leu. The full variant–phenotype cross-tabulation, together with the corresponding exploratory statistical tests, is provided in Supplementary Table S1.

### 3.6. Acquired Thrombophilia and Additional Laboratory Findings

Homocysteine was measured in 38 patients and showed a broadly similar distribution across phenotypes. Factor VIII was elevated in two patients, both with PE and ischemic stroke. HLA-B51 positivity was similarly distributed across groups, and a paroxysmal nocturnal hemoglobinuria clone was negative in nine of 11 tested. Antiphospholipid antibodies were tested inconsistently, with anti-β2-glycoprotein I requested in only 16 patients and anticardiolipin in 99. Lupus anticoagulant was positive in 18 of 130 tested, but confirmatory repeat testing was performed in only 11 and correctly timed (≥12 weeks) in only six; four patients received a new antiphospholipid syndrome diagnosis and one had a prior diagnosis. Among the 124 patients carrying at least one MTHFR c.677C>T or c.1298A>C variant, homocysteine was measured in only 35 (28.2%) and was elevated in eight of these 35 patients (22.9%).

### 3.7. Appropriateness of Natural Anticoagulant Testing

**Figure 2:**
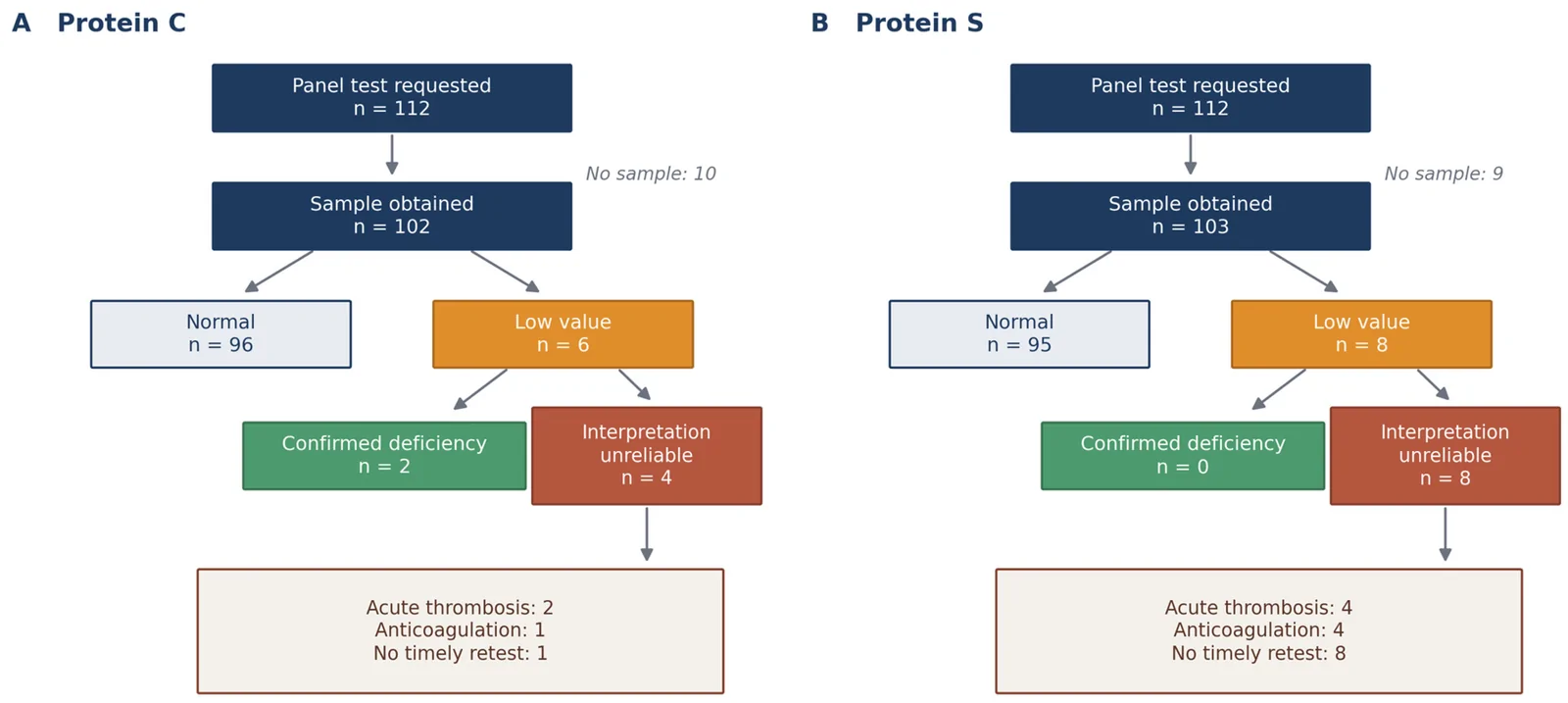
Flow of protein C (**A**) and protein S (**B**) testing and interpretation. Only protein C yielded confirmed deficiencies; every low protein S value was obtained under conditions that preclude reliable interpretation (acute thrombosis, anticoagulation, or absence of a timely confirmatory retest). Values denote the number of patients at each step.

Overall, 42 tests (32.0%) were ordered during acute thrombosis. Protein C was low in six of 102 sampled patients, but four were obtained under interpretation-limiting conditions, leaving two confirmed deficiencies. Protein S was low in eight of 103, all obtained during acute thrombosis, anticoagulation, or without timely confirmation, leaving no reliably confirmed deficiency (Figure 2).

### 3.8. Appropriateness of Panel Requests

**Figure 3:**
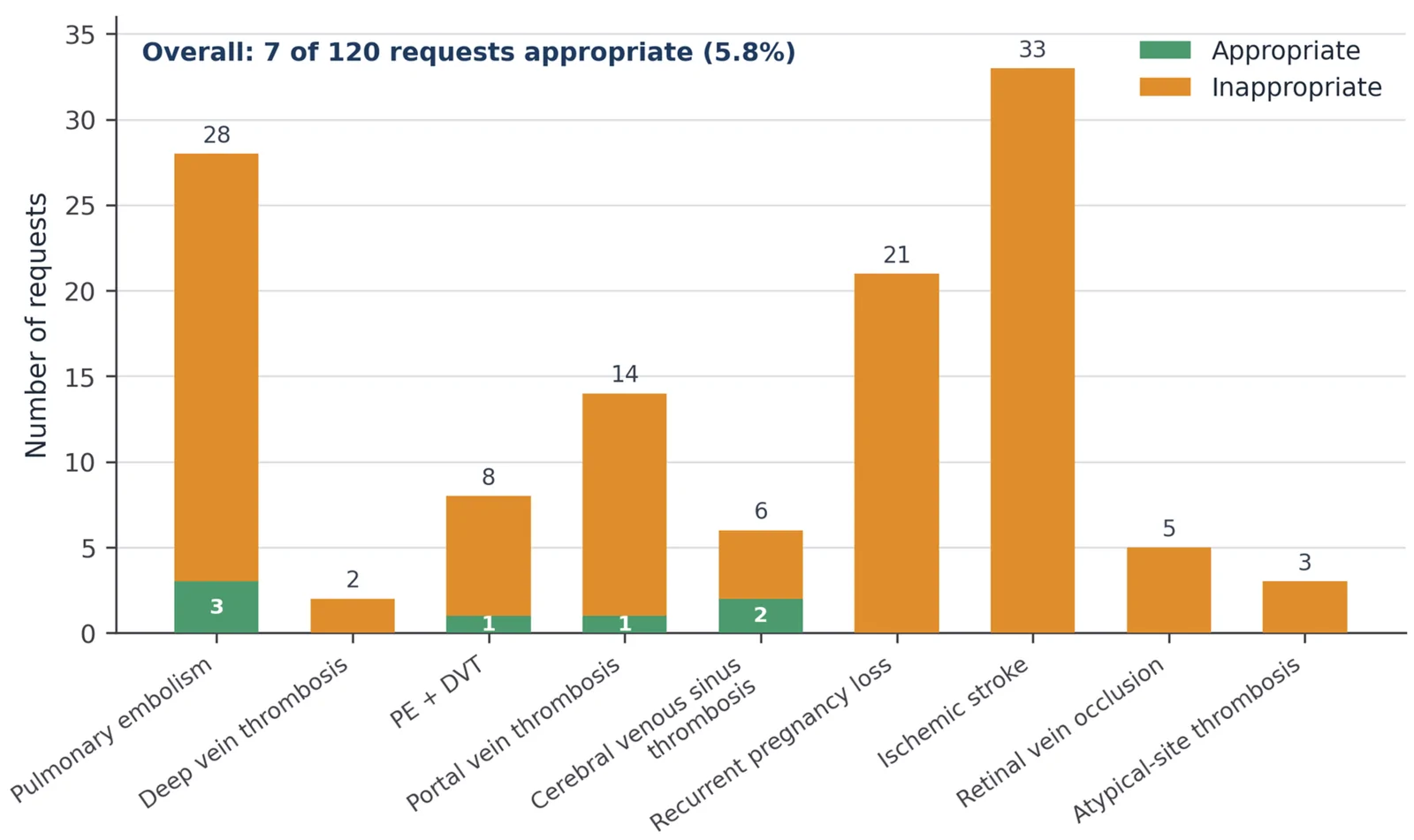
Appropriateness of hereditary thrombophilia panel requests by indication, assessed against the 2011 Turkish Society of Hematology guideline (120 evaluable requests). Only 7 requests (5.8%) met an accepted indication. PE, pulmonary embolism; DVT, deep vein thrombosis.

**Figure 4:**
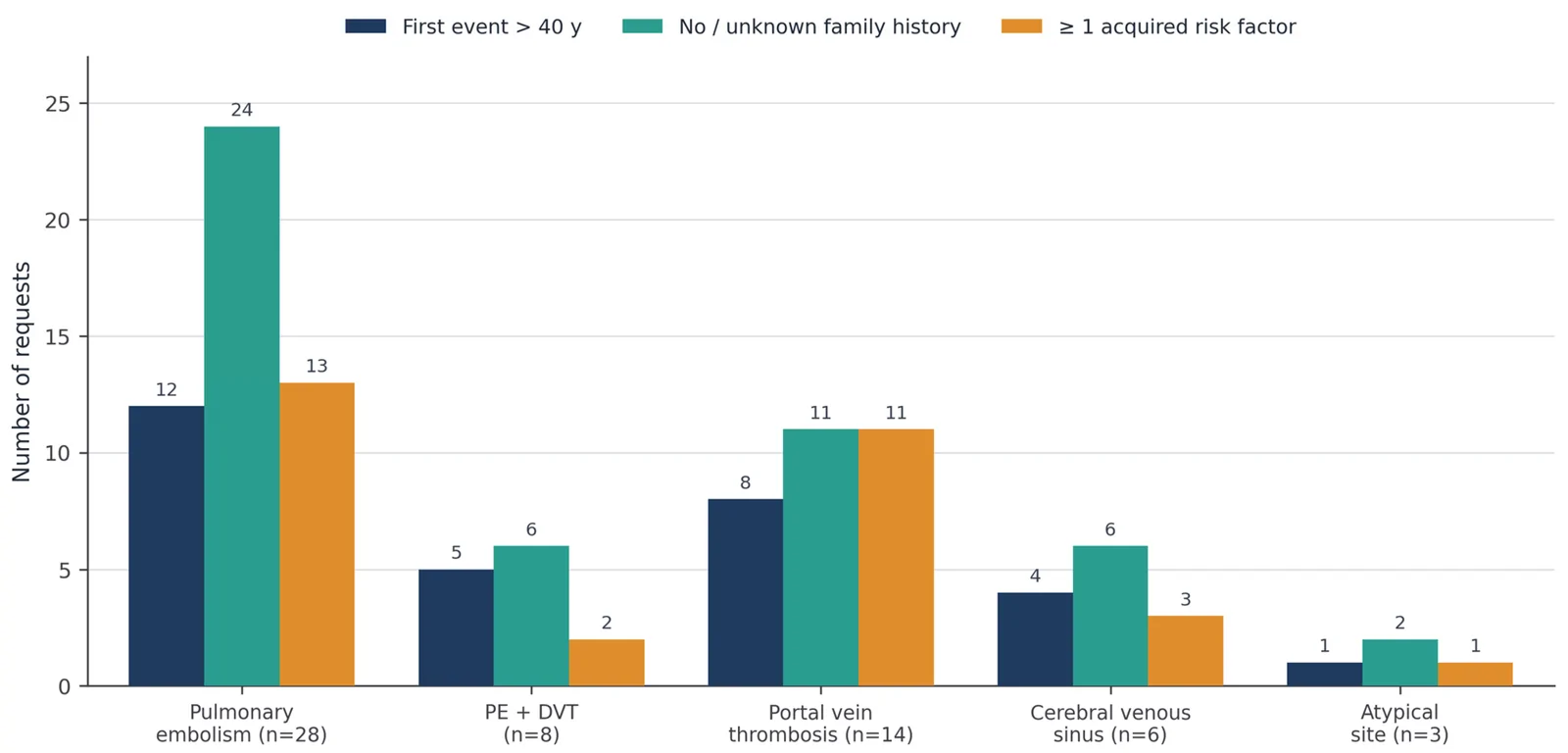
Reasons for inappropriate testing, by indication, among evaluable venous and unusual-site requests. Categories are not mutually exclusive; a single request could be inappropriate for more than one reason.

Of 120 evaluable requests, only seven (5.8%) met an accepted indication (Figure 3); the remaining 94.2% were inappropriate, most often owing to absent family history, a first event after age 40, arterial thrombosis, RPL or retinal vein occlusion, or acquired provoking factors (Figure 4). The panel had been repeated in 13 patients (9.9%).

## 4. Discussion

In this real-world cohort of adults referred to hematology after a completed thrombophilia panel, only seven of 120 evaluable requests (5.8%) fulfilled the national guideline in force during the study period. Ischemic stroke, PE, and RPL accounted for almost two-thirds of requests, ordered across 15 specialties. Beyond this low concordance, test selection, timing, and confirmation were frequently flawed: nearly one-third of patients were tested during acute thrombosis, most low protein C or S results were uninterpretable, and few positive lupus anticoagulant results underwent timely confirmation. The genetic findings contrasted established variants with low-value polymorphisms—FVL tracked with venous manifestations, whereas MTHFR, PAI-1, PGM, and most factor XIII results showed no coherent phenotype associations.

Our 5.8% appropriateness rate is lower than most prior estimates but directionally consistent with widespread overuse; previous studies report inappropriate testing in roughly 37% to 90% of cases, depending on population, setting, and criteria [4,5,6,16,17,18]. Direct comparison is difficult, since studies variably assessed assays or panels, often only in inpatients. The message is consistent: testing is often initiated without asking whether the result will alter management. Our cohort also comprised only the 131 of 554 tested adults (23.6%) referred to hematology, a referral-selected subgroup whose variant frequencies and yield need not match those of an unselected testing population.

The very low appropriateness reflected several overlapping factors rather than a single error—arterial thrombosis, RPL or retinal vein occlusion, absent family history, a first event after 40 years, and acquired provoking factors such as malignancy or immobilization—mirroring scenarios described in consensus guidance [3,4,5,6,16,17,18]. The involvement of 15 specialties indicates a problem not confined to one discipline, reflecting a fixed panel used as a general etiologic screen rather than a targeted investigation tied to management.

Ischemic stroke, the commonest indication (25.2%), is notable because inherited thrombophilias are principally venous. Although meta-analyses report associations with arterial stroke, their clinical implications are uncertain, and routine testing is not recommended even in young adults with stroke [7]. Our data reinforce this: heterozygous FVL was associated with PE and DVT but was less frequent in stroke than in non-carriers, likely reflecting the venous specificity of FVL and referral-related selection rather than protection against stroke. In stroke, targeted evaluation for antiphospholipid syndrome is generally more relevant than inherited genotyping [7].

RPL was the third most frequent indication (16.0%), yet no panel variant was associated with it, consistent with recommendations against routine inherited thrombophilia screening for recurrent miscarriage. Although some observational studies suggest associations, the evidence is heterogeneous, and the ALIFE2 trial showed no improvement in live-birth rates with low-molecular-weight heparin in this setting [8]. Testing may remain reasonable with a personal VTE history or strong family history, but RPL alone should not trigger a fixed panel.

Among variants, FVL produced the clearest, biologically coherent signals—PE in 48.6% of heterozygous carriers versus 22.1% of non-carriers, and DVT in 28.6% versus 9.5%—consistent with its established VTE association [1,9]. PGM showed no apparent association, but with only 19 carriers this reflects limited power rather than absence of effect. Recurrence showed no clear excess among heterozygous FVL carriers, consistent with guidance that common heterozygous variants alone do not determine anticoagulation duration. In the RIETE registry of more than 100,000 patients, anticoagulant selection was not materially altered by inherited thrombophilia testing [19]; management remains driven by whether the event was provoked, recurrence and bleeding risk, and patient preference [1,2,3,19].

MTHFR c.677C>T and c.1298A>C were common but showed no consistent phenotype relationship; the isolated DVT difference across c.677C>T genotypes lost significance on pairwise testing and, given multiple comparisons, warrants caution. Large pooled analyses show no consistent independent association between MTHFR polymorphisms and VTE [9]. Similarly, PAI-1 4G/5G—the most frequent result—was not associated with any manifestation; the 4G allele confers, at most, a small effect, and neither PAI-1 nor factor XIII genotyping has a validated role [10,11,12]. The factor XIII-CVST signal rested on only five homozygous individuals and is hypothesis-generating, not a basis for routine testing. These markers were examined only because they remained bundled in the locally available panel, a feature that itself illustrates the low-value testing practice evaluated in this study. MTHFR, PAI-1, and factor XIII polymorphisms have no validated role in routine thrombophilia assessment and are not included among recommended heritable thrombophilia tests in contemporary international guidance; their distributions are therefore reported descriptively, and the related comparisons—several involving very small subgroups—should be regarded as exploratory and hypothesis-generating rather than confirmatory. Among the 124 patients carrying at least one MTHFR variant, homocysteine was measured in only 35 (28.2%) and was elevated in eight (22.9%); however, common MTHFR polymorphisms are not established risk factors for arterial or venous thrombosis, and MTHFR genotyping has no validated role irrespective of homocysteine concentration.

Timing errors compromised natural anticoagulant testing: of six low protein C results only two were confirmed, and all eight low protein S results were obtained during acute thrombosis, anticoagulation, or without timely confirmation, leaving none valid. Acute thrombosis, inflammation, pregnancy, liver dysfunction, and anticoagulants alter these levels [3,20,21,22]; an abnormal acute-phase result therefore requires confirmation after treatment effects resolve [22]. Anticoagulant interference similarly affects lupus anticoagulant assays [20,21]: only 33.3% of initially positive patients underwent correctly timed confirmation, close to another single-center report [23] but well below the roughly 77% in a large administrative database [24]. An initial positive result is not equivalent to antiphospholipid syndrome, and persistence must be demonstrated. Despite fragmented antibody profiles, four new syndrome diagnoses were established, showing that appropriately selected acquired testing yields meaningful findings, unlike indiscriminate panels. In our laboratory, lupus anticoagulant was assessed using a single dRVVT-based method; the absence of a complementary aPTT-based assay may have reduced sensitivity relative to the two-system approach recommended by contemporary ISTH-SSC guidance, which also details the associated preanalytical and interpretive pitfalls [25].

These findings carry stewardship implications. Fixed panels bundle established thrombophilias, low-value polymorphisms, and assays requiring different timing, whereas targeted testing is likely more informative and less costly [26]; local guidance can markedly reduce ordering, with an 84% reduction reported after implementation of a local guideline [4]. A practical strategy would remove MTHFR, PAI-1, and factor XIII from routine panels, restrict natural anticoagulant assays during acute thrombosis or interfering anticoagulation, mandate documentation of indication and treatment status, and reserve testing for reflexive or specialist-approved scenarios.

At our institution, thrombophilia-panel ordering was not subject to hematology or laboratory gatekeeping during the study period. Although this audit did not lead to an immediate formal modification of the ordering pathway, it prompted the development of a diagnostic-stewardship proposal that is now under institutional review. The proposed measures include removal of MTHFR, PAI-1, and factor XIII from the bundled panel; an electronic block on redundant repeat genetic testing; mandatory documentation of the clinical indication and anticoagulant status at ordering; and hematology or laboratory review of selected requests. Anticipated barriers include the convenience of a single bundled order, entrenched ordering habits across multiple specialties, and a low reimbursed per-panel tariff (approximately US$21 at study-period exchange rates), which provides little financial deterrent; durable change will therefore require electronic order-set redesign, institutional implementation, and targeted clinician education rather than reliance on cost considerations alone.

Strengths include consecutive referrals from multiple specialties, evaluation of indication, timing, and confirmation rather than genetics alone, and a predefined national standard for appropriateness. Limitations include the retrospective single-center design, referral and selection bias precluding prevalence or absolute-risk estimates, absence of a control group, very small subgroups, multiple unadjusted comparisons, incomplete ancillary testing, and use of a national guideline that differs from newer international recommendations. Downstream effects on anticoagulation duration, anxiety, referrals, outcomes, and cost were not assessed.

## 5. Conclusions

Hereditary thrombophilia panels are predominantly ordered outside guideline-supported indications, most often in settings—ischemic stroke and RPL—where their utility is limited. In our study, FVL showed the expected venous association, whereas common MTHFR and PAI-1 polymorphisms offered no coherent discriminatory information, and the factor XIII-CVST signal required cautious interpretation. Errors in timing and confirmation markedly reduced the validity of protein C, protein S, and lupus anticoagulant results. Fixed, indiscriminate panels should be replaced by targeted testing guided by clinical phenotype, likely impact on management, anticoagulant exposure, and appropriate confirmation.

## Figures and Tables

**Table 1:** Institutional laboratory reference ranges used for the interpretation of additional thrombophilia testing.

Parameter	Unit	Reference Range
Lupus anticoagulant	ratio	0.9–1.20
Factor VIII	IU/dL	70–150
Homocysteine	µmol/L	5–15

Lupus anticoagulant is expressed as a normalized clotting-time ratio. IU/dL, international units per deciliter.

**Table 2:** Screening recommendations of the 2011 Turkish Society of Hematology guideline on hereditary thrombophilia.

Recommendation	Clinical Situation
S creening recommended	Family predisposition to thrombosis, or a first venous thromboembolism before 40 years of age; purpura fulminans (screen especially for protein C and protein S deficiency).
Screening may be considered	Cerebral or intra-abdominal vein thrombosis (for intra-abdominal thrombosis, exclude acquired causes first); family screening in thrombosis-prone families when a positive result would change management, and for high-risk thrombophilic disorders.
Screening not recommended	Upper-extremity thrombosis (thoracic outlet- or catheter-related) and retinal vein occlusion; major transient risk factors, active cancer, or other predisposing conditions (e.g., SLE, inflammatory bowel disease, myeloproliferative neoplasm); routine screening of hospitalized medical inpatients; patients and family members older than 60 years; children who have had a stroke; family screening for low-risk rare homozygous or compound heterozygous variants.

Summarized from the 2011 Turkish Society of Hematology guideline [15], the national standard in force during the study period; used as the reference standard for the appropriateness analysis. More recent international guidance is considered in the Discussion. SLE, systemic lupus erythematosus.

**Table 3:** Baseline demographic and clinical characteristics of the study population.

Characteristic	Value (*n* = 131)
Age at first thrombotic event, mean ± SD (range), years	39.7 ± 11.6 (18–70)
≤40 years	72 (55.0)
>40 years	59 (45.0)
Female sex	84 (64.1)
Male sex	47 (35.9)
Hyperlipidemia	30 (22.9)
Obesity	28 (21.4)
Hypertension	27 (20.6)
Diabetes mellitus	18 (13.7)
Malignancy	7 (5.3)
Rheumatologic disease	5 (3.8)

Data are *n* (%) unless otherwise stated. SD, standard deviation.

**Table 4:** Demographic characteristics and comorbidities by thrombotic phenotype.

Variable	Arterial (*n* = 39)	Venous (*n* = 62)	Arterial & Venous (*n* = 7)	RPL (*n* = 21)
Female sex	27 (69.2)	31 (50.0)	4 (57.1)	—
Age, median (range), y	42 (20–56)	40 (18–70)	36 (27–52)	—
Hyperlipidemia	13 (34.2)	11 (18.6)	4 (57.1)	2 (9.5)
Diabetes mellitus	4 (10.5)	10 (16.9)	2 (28.6)	2 (10.0)
Hypertension	12 (31.6)	12 (20.0)	2 (28.6)	1 (4.8)
Rheumatologic disease	1 (2.6)	4 (6.5)	0	0
Malignancy	0	5 (8.1)	2 (28.6)	0

Data are *n* (%) unless otherwise stated; denominators vary slightly by variable owing to missing data. The RPL group was excluded from sex and age comparisons. Values are presented descriptively; formal statistical comparison was not performed for these subgroups. RPL, recurrent pregnancy loss.

**Table 5:** Acquired (secondary) risk factors by thrombotic phenotype.

Variable	Arterial (*n* = 39)	Venous (*n* = 62)	Arterial & Venous (*n* = 7)	RPL (*n* = 21)
Smoking	16 (42.1)	27 (44.3)	4 (57.1)	3 (14.3)
Obesity	7 (20.6)	15 (26.8)	4 (57.1)	2 (9.5)
Immobilization	2 (5.3)	7 (11.7)	1 (14.3)	0
Myeloproliferative neoplasm	2 (5.1)	2 (3.2)	1 (14.3)	0
Cirrhosis	0	4 (6.6)	0	0
Family history of thrombosis	2 (5.3)	11 (18.0)	1 (16.7)	2 (10.0)
Nephrotic syndrome	1 (2.6)	2 (3.3)	1 (14.3)	0
Oral contraceptive use	0	5 (8.1)	0	0
Pregnancy	2 (5.1)	4 (6.5)	0	0

Data are *n* (%); denominators vary slightly by variable owing to missing data. Values are presented descriptively. No patient was receiving hormone replacement therapy or had an indwelling catheter, and one patient was postpartum; these variables were not analyzed. RPL, recurrent pregnancy loss.

**Table 6:** Distribution of the six hereditary thrombophilia panel variants.

Variant/Genotype	*n* (%)
Factor V Leiden (*n* = 130)	
Heterozygous	35 (26.9)
Homozygous	1 (0.8)
No mutation	94 (72.3)
PAI-1 (*n* = 131)	
4G/5G	64 (48.9)
4G/4G	34 (26.0)
5G/5G	33 (25.2)
MTHFR c.677C>T (*n* = 131)	
Heterozygous	60 (45.8)
Homozygous	30 (22.9)
No mutation	41 (31.3)
MTHFR c.1298A>C (*n* = 131)	
Heterozygous	51 (38.9)
Homozygous	10 (7.6)
No mutation	70 (53.4)
F13A1 Val34Leu (V34L; rs5985) (*n* = 131)	
Heterozygous	39 (29.8)
Homozygous	5 (3.8)
No mutation	87 (66.4)
Prothrombin G20210A (*n* = 131)	
Heterozygous	18 (13.7)
Homozygous	1 (0.8)
No mutation	112 (85.5)

PAI-1, plasminogen activator inhibitor-1; MTHFR, methylenetetrahydrofolate reductase. Factor V Leiden was successfully genotyped in 130 patients.

## Data Availability

The data presented in this study are available on request from the corresponding author. The data are not publicly available owing to privacy restrictions.

## Supplementary Materials

The following supporting information can be downloaded at: https://www.mdpi.com/article/10.3390/diagnostics16162643/s1, Table S1: Variant–phenotype cross-tabulation (association between hereditary thrombophilia variant type and thrombotic manifestation), presented as exploratory analysis.

## Abbreviations

The following abbreviations are used in this manuscript:

APS	Antiphospholipid syndrome
CVST	Cerebral venous sinus thrombosis
DVT	Deep vein thrombosis
FVL	Factor V Leiden
LA	Lupus anticoagulant
MTHFR	Methylenetetrahydrofolate reductase
PAI-1	Plasminogen activator inhibitor-1
PE	Pulmonary embolism
PGM	Prothrombin G20210A variant
RPL	Recurrent pregnancy loss
VTE	Venous thromboembolism
