# Supplementary material for: Beyond the Thrombophilia Panel: Real-World Overuse, Limited Interpretability, and Poor Guideline Concordance in a Hematology Referral Cohort
Source: Diagnostics (Basel). 2026 Aug 19;16(16):2643. doi: 10.3390/diagnostics16162643 (PMC13512108; doi:10.3390/diagnostics16162643)
Supplement: Supplementary file 1 [file diagnostics-16-02643-s001.zip › diagnostics-4456380-supplementary.pdf]

Article

Beyond the Thrombophilia Panel: Real-World Overuse, Limited Interpretability, and Poor Guideline Concordance in a Hematology Referral Cohort

Aysenur Ozturk Ari[1], Ibrahim Ethem Pinar[2,*], Vildan Ozkocaman[2], Fahir Ozkalemkas[2]

[1] *Department of Internal Medicine, Faculty of Medicine, Bursa Uludag University, Bursa 16059, Turkiye; dr.aysenurozturk@gmail.com*
[2] *Department of Hematology, Faculty of Medicine, Bursa Uludag University, Bursa 16059, Turkiye*

[*] Correspondence: iethempinar@uludag.edu.tr

Table S1. Association between hereditary thrombophilia variant type and thrombotic manifestation (exploratory analysis).

Thrombotic manifestation	Factor V Leiden				PAI-1				MTHFR c.677C>T			
	Het	Homo	None	p	4G/5G	4G/4G	5G/5G	p	Het	Homo	None	p
Pulmonary embolism	17 (48.6)	1 (100)	21 (22.1)	**0.003**	22 (34.4)	8 (23.5)	9 (27.3)	0.501	22 (36.7)	6 (20.0)	11 (26.8)	0.234
Deep vein thrombosis	10 (28.6)	1 (100)	9 (9.5)	**0.003**	7 (10.9)	5 (14.7)	8 (24.2)	0.224	15 (25.0)	2 (6.7)	3 (7.3)	**0.017**
Ischemic stroke	4 (11.4)	0	32 (33.7)	**0.017**	15 (23.4)	10 (29.4)	11 (33.3)	0.561	12 (20.0)	9 (30.0)	15 (36.6)	0.175
Cerebral venous sinus thrombosis	0	0	6 (6.3)	0.227	3 (4.7)	3 (8.8)	0	0.231	3 (5.0)	2 (6.7)	1 (2.4)	0.764
Recurrent pregnancy loss	12 (34.3)	0	19 (20.0)	0.195	13 (20.3)	10 (29.4)	8 (24.2)	0.598	13 (21.7)	7 (23.3)	11 (26.8)	0.835
Portal vein thrombosis	1 (2.9)	0	15 (15.8)	0.152	6 (9.4)	6 (17.6)	4 (12.1)	0.541	7 (11.7)	3 (10.0)	6 (14.6)	0.835
Retinal vein occlusion	1 (2.9)	0	3 (3.2)	>0.999	3 (4.7)	1 (2.9)	0	0.807	2 (3.3)	2 (6.7)	0	0.266
Atypical-site thrombosis	1 (2.9)	0	5 (5.3)	>0.999	3 (4.7)	2 (5.9)	1 (3.0)	>0.999	4 (6.7)	0	2 (4.9)	0.480
Thrombosis recurrence (≥2 events)	12 (34.3)	1 (100)	19 (20.0)	**0.008**	14 (21.9)	9 (26.5)	9 (27.3)	0.803	19 (31.7)	3 (10.0)	10 (24.4)	0.066

Thrombotic manifestation	MTHFR c.1298A>C				F13A1 Val34Leu				Prothrombin G20210A			
	Het	Homo	None	p	Het	Homo	None	p	Het	Homo	None	p
Pulmonary embolism	19 (37.3)	4 (40.0)	16 (22.9)	0.177	14 (35.9)	0	25 (38.7)	0.299	6 (33.3)	0	33 (29.5)	0.848
Deep vein thrombosis	7 (13.7)	2 (20.0)	11 (15.7)	0.870	7 (17.9)	0	13 (14.9)	0.826	4 (22.2)	0	16 (14.3)	0.558
Ischemic stroke	13 (25.5)	4 (40.0)	19 (27.1)	0.640	10 (25.6)	3 (60.0)	23 (26.4)	0.290	6 (33.3)	0	30 (26.8)	0.693
Cerebral venous sinus thrombosis	2 (3.9)	1 (10.0)	3 (4.3)	0.631	2 (5.1)	2 (40.0)	2 (2.3)	**0.011**	0	0	6 (5.4)	0.614

Thrombotic manifestation	Factor V Leiden				PAI-1				MTHFR c.677C>T			
	Het	Homo	None	p	4G/5G	4G/4G	5G/5G	p	Het	Homo	None	p
Recurrent pregnancy loss	13 (25.5)	3 (30.0)	15 (21.4)	0.775	10 (25.6)	2 (40.0)	19 (21.8)	0.523	6 (33.3)	1 (100)	24 (21.4)	0.140
Portal vein thrombosis	7 (13.7)	1 (10.0)	8 (11.4)	0.907	4 (10.3)	1 (20.0)	11 (12.6)	0.684	2 (11.1)	0	14 (12.5)	>0.999
Retinal vein occlusion	0	0	4 (5.7)	0.269	1 (2.6)	0	3 (3.4)	>0.999	1 (5.6)	0	3 (2.7)	0.470
Atypical-site thrombosis	2 (3.9)	0	4 (5.7)	>0.999	0	0	6 (6.9)	0.351	0	0	6 (5.4)	0.614
Thrombosis recurrence (≥2 events)	13 (25.5)	4 (40.0)	15 (21.4)	0.797	7 (17.9)	2 (40.0)	23 (26.4)	0.836	6 (33.3)	0	26 (23.2)	0.180

Data are n (%). Because several genotype subgroups were very small, these variant–phenotype comparisons are exploratory and hypothesis-generating rather than confirmatory; p values (chi-square or Fisher–Freeman–Halton test) are provided for completeness only. Het, heterozygous; Homo, homozygous; None, no mutation; PAI-1, plasminogen activator inhibitor-1; MTHFR, methylenetetrahydrofolate reductase.
